# ECMO in cardiac arrest and cardiogenic shock

**DOI:** 10.1007/s00059-016-4523-4

**Published:** 2017-01-26

**Authors:** L. C. Napp, C. Kühn, J. Bauersachs

**Affiliations:** 1 Cardiac Arrest Center, Acute and Advanced Heart Failure Unit, Department of Cardiology and Angiology, Hannover Medical School, Carl-Neuberg-Str. 1, 30625 Hannover, Germany; 2 Department of Cardiothoracic, Transplantation and Vascular Surgery, Hannover Medical School, Hannover, Germany

**Keywords:** Cardiogenic shock, Cardiac arrest, Sudden cardiac death, Cardiopulmonary resuscitation, ECMO, Mechanical circulatory support, Microaxial pump, Extracorporeal resuscitation, Kardiogener Schock, Herz-Kreislauf-Stillstand, Plötzlicher Herztod, Wiederbelebung, ECMO, Mechanische Kreislaufunterstützung, Mikroaxialpumpe, Extrakorporale Reanimation

## Abstract

Cardiogenic shock is an acute emergency, which is classically managed by medical support with inotropes or vasopressors and frequently requires invasive ventilation. However, both catecholamines and ventilation are associated with a worse prognosis, and many patients deteriorate despite all efforts. Mechanical circulatory support is increasingly considered to allow for recovery or to bridge until making a decision or definite treatment. Of all devices, extracorporeal membrane oxygenation (ECMO) is the most widely used. Here we review features and strategical considerations for the use of ECMO in cardiogenic shock and cardiac arrest.

## Zusammenfassung

Der kardiogene Schock ist ein akut lebensbedrohlicher Notfall, der klassischerweise medikamentös (u. a. Inotropika und ggf. Vasopressoren) behandelt wird und häufig eine invasive Beatmung erfordert. Katecholamine und Beatmung sind jedoch mit einer ungünstigen Prognose assoziiert, und viele Patienten sind mit konservativen Maßnahmen nicht zu stabilisieren. Mechanische Kreislaufunterstützung wird immer öfter herangezogen, um den Kreislauf zu stabilisieren, dem erkrankten Herzen Zeit zur Erholung zu verschaffen oder eine Überbrückung bis zur definitiven Therapie zu etablieren. Das aktuell weltweit am häufigsten eingesetzte System zur mechanischen Kreislaufunterstützung in diesem Zusammenhang ist die extrakorporale Membranoxygenierung (ECMO). In der vorliegenden Übersicht fassen die Autoren die speziellen Eigenschaften dieses Systems sowie strategische Überlegungen im Kontext des kardiogenen Schocks und des Herz-Kreislauf-Stillstands zusammen.

Cardiogenic shock and cardiac arrest are life-threatening emergencies with a high mortality rate despite numerous efforts in diagnosis and therapy. For a long time medical therapy – at the forefront with catecholamines, vasodilators and others – and mechanical ventilation, if necessary, were the standard of care for cardiogenic shock. Oxygen supply and perfusion are critically reduced during shock and arrest, and both are physical processes that are in principle amenable to (temporary) extracorporeal mechanical support. Early pioneering work to prove this principle was performed in animals as early as 1937 [1] and in humans 20–30 years later [2, 3]. With the seminal paper by Hill and coworkers [4], extracorporeal membrane oxygenation (ECMO), which can provide blood flow support and extracorporeal gas exchange at the same time, was introduced into the clinic. Since then, technical improvements have contributed to the current worldwide use of ECMO for severe respiratory and cardiorespiratory failure refractory to medical therapy. Recently, there has been some discussion on initiating mechanical support even earlier, with the intention to avoid multiorgan failure associated with excessive catecholamine doses and/or aggressive ventilator settings. By analogy with the concept of veno-venous ECMO and lung-protective ventilation for treatment of acute respiratory distress syndrome, the goal of mechanical support in cardiogenic shock is myocardial rest while protecting end organ perfusion.

In the following, we review ECMO support in the context of cardiogenic shock and refractory cardiac arrest, with a special focus on technical aspects of veno-arterial ECMO. Of note, the following statements are primarily true for percutaneous ECMO with femoral cannulation and may not necessarily be directly transferable to central or upper-body cannulation.

## Cardiogenic shock and cardiac arrest

Cardiogenic shock is the main cause of early mortality in patients with acute myocardial infarction [5]. Other conditions leading to shock comprise acutely decompensated chronic heart failure, decompensated valvular heart disease, myocarditis, Takotsubo syndrome, acute pulmonary embolism, acute allograft failure, incessant arrhythmia, peripartum cardiomyopathy [6], and others [7]. During cardiogenic shock not only the heart itself suffers from pump failure, but even more end organs such as the brain, kidney, liver, and gut are at risk due to insufficient perfusion (multiorgan dysfunction syndrome) [8], and the rate of congestion-associated pneumonia increases. Beyond blood pressure and heart rate as classic shock markers, serum lactate, central venous oxygenation, liver enzyme levels, and urine output are surrogate markers of circulatory failure and multiorgan dysfunction [9]. Reduced coronary perfusion further decreases cardiac output, and multiorgan dysfunction/failure is further complicated by metabolic acidosis and acute coagulopathy. All of these conditions aggravate each other in a fatal vicious circle [8, 9].

Out-of-hospital cardiac arrest (OHCA) occurs with an estimated incidence of 500,000 per year in Europe [10, 11], with two thirds having a primary cardiac cause [12]. Mortality after OHCA remains high despite interventional therapy and modern intensive care. Only 10–15% of those who arrive at the normal hospital survive [13, 14], of whom about 50–80% have a favorable neurological prognosis [15, 16]. In this context, immediate bystander CPR and area-wide availability of automated external defibrillators are essential to increase survival and prognosis. The first electric shock should be applied as early as possible [17] to minimize the time of hypoperfusion, associated LV pump failure, and consecutive development of shock [18]. After return of spontaneous circulation (ROSC), the patient needs to be transferred to an experienced center, which holds all required diagnostic and therapeutic tools [19]. In clinical routine, the first 24 h after resuscitation often decide on the outcome, and guidelines recommend cardiac catheterization in most cases early after OHCA [20, 21]. Therefore, primary admission to a tertiary center should be preferred over admission to a regional hospital and secondary transfer to a tertiary center, when progression of shock has already occurred.

The majority of patients after OHCA develop post-cardiac arrest syndrome [22, 23] in a vicious circle: Cardiac arrest leads to ischemia of the myocardium and end organs, which results in adverse metabolism, acidosis, and vasoplegia. The hypoperfused heart is not able to respond to the circulatory needs, which in turn aggravates peripheral ischemia [24]. Therefore, restoration of systemic perfusion is essential – particularly in the immediate and early phase after ROSC – in order to limit multiorgan dysfunction [25], which can also be considered a “whole-body reperfusion syndrome.” In this context, complete cardiac revascularization is recommended [12, 26], but care of other end organs such as the brain, intestine, liver, and kidneys is equally important [23].

As outlined, cardiogenic shock and cardiac arrest share many pathophysiological features and evoke many similar responses. Thus, it was not surprising but very important to prove that the prognosis of both conditions is equally adverse: In a recent study of 250 consecutive patients from Denmark, 130 were admitted to a tertiary center with cardiogenic shock, while 118 had OHCA. Interestingly, both groups had the same dismal outcome with 60% 1‑week mortality [27]. This underlines the urgent need for novel therapeutic strategies for patients with cardiogenic shock and arrest.

## Restoration of systemic circulation

For many years catecholamines have been used for stabilization of patients with cardiogenic shock. Inotropes such as dobutamine are given with the intention to increase cardiac output by their positive inotropic and chronotropic function. In contrast, vasopressors such as norepinephrine are administered for increasing blood pressure by vasoconstriction and indirect effects such as increased preload. Epinephrine shares features of both drug classes. However, inotropic drugs increase myocardial oxygen consumption, heart rate, arrhythmogenicity, and inflammation in the already diseased heart [28]. Beta1-adrenoceptor agonists have been associated with energy depletion, oxidative stress, and adverse outcome in acute heart failure [29]. Vasopressors increase myocardial afterload and potentially impair peripheral tissue perfusion. Thus, from a pathophysiological perspective, inotropes as well as vasopressors are associated with adverse effects on the heart and other end organs while these organs should recover. Consistently, current guidelines recommend catecholamines as a short-term bridge in the acute situation (only class IIb, level of evidence C), but clearly mention the disadvantages of such drugs, also in light of the paucity of clinical studies demonstrating a survival benefit [25, 30, 31]. In clinical routine, catecholamines are often “effective” in terms of increasing blood pressure, but linked to impaired microcirculation and multiorgan failure, and thus not sufficient for sustained and harmless stabilization of patients with severe cardiogenic shock and resuscitation. In this context, beta-blockers and calcium antagonists taken by the patient before arrest might further contribute to the limited efficacy of catecholamines.

Therefore, it is increasingly being discussed to initiate mechanical circulatory support as a powerful tool for bridging earlier and more frequently, in order to improve the prognosis of patients with severe cardiogenic shock or refractory arrest [32]. However, this trend is based on data from many registries and retrospective/observational studies, while evidence from prospective randomized controlled studies is lacking.

## Mechanical circulatory support

Several modes and devices of mechanical support are currently available [32], of which each has its own features and advantages.

The intra-aortic balloon pump (IABP) consists of a catheter-mounted balloon that inflates during diastole and deflates during systole in the descending thoracic aorta. By this, coronary perfusion should be enhanced during diastole, while afterload should be decreased during systole when the left ventricle (LV) ejects. Notwithstanding the attractive pathophysiological principle, augmentation by IABP depends on LV output, and the potential of support decreases with lower LV output. Several studies have demonstrated that IABP support is not favorable in infarct-related cardiogenic shock [33, 34]. Therefore, current guidelines have retracted the recommendation of IABP use [31].

The TandemHeart® consists of a pump and two cannulas, of which one is inserted via venous access and transseptal approach into the left atrium (LA), and the other one via arterial access into the femoral artery. By this, the TandemHeart® introduces a right-to-left shunt, reduces LV preload by LA drainage, but increases afterload by retrograde flow support toward the aorta. The TandemHeart® is not widely used in Europe and requires experienced transseptal cannula placement, which is assumed to harbor considerable risk in the acute situation.

Transaortic microaxial pumps (Impella®, Heartmate PHP®) are introduced via arterial access through the aorta across the aortic valve into the LV. These devices directly unload the LV, transport the drained volume inside of the pump toward the aorta and eject into the aortic root. This elegant approach, which follows the physiological blood flow direction, is comprehensively described in the same issue of this journal (Schäfer A, Bauersachs J, doi: 10.1007/s00059-016-4512-7). However, microaxial pumps do not offer gas exchange or temperature control.

Probably, the most often used form of mechanical circulatory support today is ECMO. Originating from cardiac surgery and initially developed for temporary lung replacement, ECMO support is now broadly established for cardiorespiratory support [35]. Notwithstanding its enormous support potential, ECMO has several special features and harbors certain specific risks, which will be reviewed here (see next sections).

In general, mechanical support can be used with different strategies (Table 1). In patients with severe cardiogenic shock from myocardial infarction or myocarditis, mechanical support is routinely employed in a *bridge-to-recovery* approach. In the case of acute decompensated chronic heart failure, the potential for recovery may be limited, which sometimes results in a *bridge-to-destination* approach. In resuscitated patients, a *bridge-to-decision* strategy is usually required, as further therapies such as LVAD surgery, ICD implantation etc. are postponed until awakening of the patient allows for estimating neurological recovery and eligibility.

**Table 1:** Strategies of mechanical circulatory support

Strategy	Indication (examples)	Principle	Goal
Bridge-to-recovery	Acute heart failure (myocarditis, acute myocardial infarction)	Stabilize systemic circulation, ensure end organ perfusion and reduce preload until myocardial recovery	Recovery
Bridge-to-transplantation	Terminal heart failure	Stabilize systemic circulation, ensure end organ perfusion until heart transplantation	Transplantation
Bridge-to-destination	Terminal heart failure	Stabilize systemic circulation, ensure end organ perfusion until LVAD implantation	LVAD
Bridge-to-surgery	Acute pulmonary embolism with shock (and contraindication for fibrinolysis)	Reduce preload and stabilize systemic circulation until emergent embolectomy	Embolectomy
Bridge-to-decision	Extracorporeal CPR	Stabilize systemic circulation, ensure end organ perfusion until (neurological) re-evaluation and decision on therapeutic strategy	Re-evaluation
	Refractory cardiogenic shock	ECMO implantation at the referral center by the ECMO team and transport to the tertiary center for further therapy	Transfer

*CPR* cardiopulmonary resuscitation, *ECMO* extracorporeal membrane oxygenation, *LVAD* left ventricular assist device

## Veno-arterial ECMO

### Technical aspects

ECMO is a modified form of cardiopulmonary bypass [36], and has undergone a dramatic technical evolution since the widely known publication by Hill and coworkers in 1972 [4]. In principle, ECMO drains venous blood through a cannula and tubing and returns it via another tubing and cannula into the body, both driven by a rotor unit. During ECMO passage the blood becomes oxygenated, decarboxylated, and warmed in an extracorporeal gas exchange unit. In nonsurgical application in adults, peripheral cannulation of the femoral and/or jugular vessels is the standard technique, usually with 21–25 French draining and 15–19 French returning cannulas (Table 2). Veno-venous (VV) ECMO drains from and returns to the right atrium. It is used for replacement of lung function, typically during acute respiratory distress syndrome, and is not further discussed here.

**Table 2:** Technical features of VA-ECMO

Implantation	Cannulation of femoral artery (15–19 Fr) and vein (21–15 Fr) with modified Seldinger’s technique takes about 10 min until circuit starts
Mobility	Inter- and intrahospital transfer, up to air-bridge (flight transfer)
Hemodynamic effect	Increased systemic perfusion by retrograde flow support
	Preload reduction
	Afterload increase
Flow rates	Up to 7 l/min, depending on cannulas and rotor/oxygenator
Gas exchange	Highly efficient oxygenation and decarboxylation of reinfused blood
Contraindications	Ethical considerations, patient’s will
	No perspective of a bridging strategy
	Severe peripheral artery disease (iliac)
	(Severe) aortic regurgitation
	Aortic dissection
	Left ventricular thrombus (relative)
	Uncontrolled bleeding disorder (relative)
Potential complications	Leg ischemia
	Bleeding
	Vascular complications
	Two-circulation syndrome
	LV distension
	Hyperfibrinolysis
	Embolism

*Fr* French, *VA-ECMO* veno-arterial extracorporeal membrane oxygenation

**Fig. 1:**
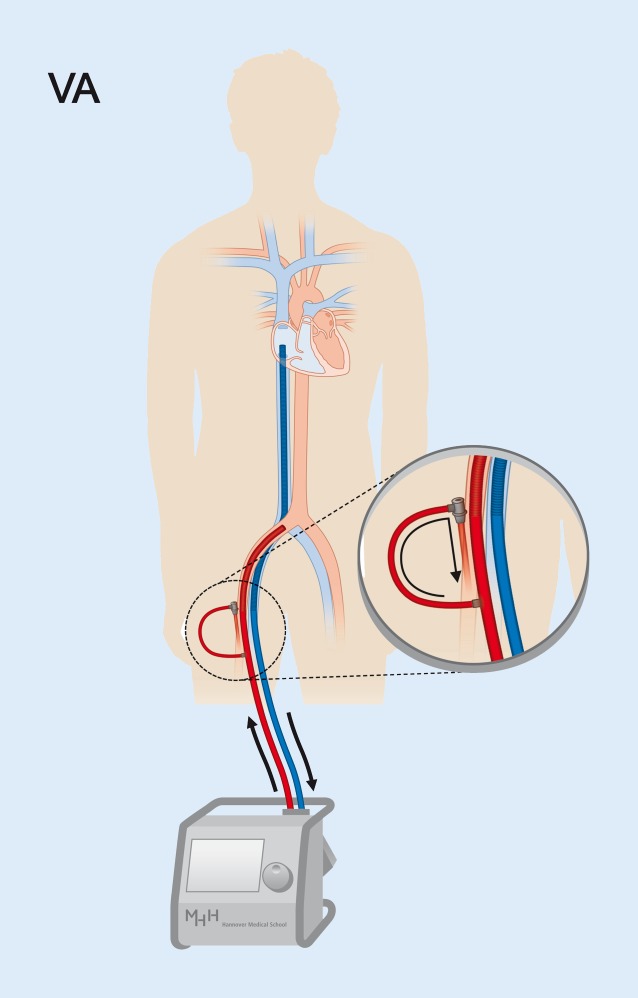
Veno-arterial (*VA*) ECMO. VA-ECMO drains venous blood (*blue*) from the right atrium and returns an equal volume after reoxygenation and decarboxylation (*red*) to the iliac artery toward the aorta. Note the position of the draining venous cannula tip in the mid right atrium. Femoral arterial cannulation requires an extra sheath for antegrade perfusion of the leg (*inset*). (Modified from Napp & Bauersachs [49]; © L. C. Napp, J. Bauersachs 2016. This publication is an open access publication, available on intechopen.com)

In contrast, veno-arterial (VA) ECMO drains blood from the right atrium and returns to the arterial system, typically to the iliac arteries toward the aorta (Fig. 1). By this, VA-ECMO reduces preload and increases aortic flow and end organ perfusion [36]. With arterial cannulation, placement of a dedicated sheath for antegrade perfusion of the cannulated leg (Fig. 1) is recommended to prevent leg ischemia [37], which is standard in many centers.

A great advantage of VA-ECMO is that cannulation may be performed nearly everywhere, as the system and all parts are transportable. Thus, an unstable patient can receive ECMO support in the emergency room, on the ward, in the catheterization laboratory, the operating theater, or even in the field [38, 39]. In contrast to other support systems, fluoroscopy or echocardiography guidance is – albeit helpful – not required for successful implantation. Once ECMO is running, the patient can be transferred with the whole unit, which is another advantage over other systems. Therefore ECMO is frequently used for transport of unstable patients by car, helicopter, or even by plane as an air-bridge [40].

VA-ECMO establishes a massive right-to-left shunt by draining venous blood and returning it to the iliac artery. This flow support, which can reach 7 l/min with large cannulas and contemporary rotors, results in a significant increase in blood pressure as long as there is enough vascular resistance (pressure = flow × resistance). The massive venous drainage effectively reduces preload and thus leads to venous decongestion. Arterial reinfusion to the systemic circulation strongly enhances perfusion of end organs and is therefore attractive during severe cardiorespiratory failure or resuscitation. Of note, at the same time retrograde flow support increases LV afterload (see next section).

### Contraindications and complications

Notwithstanding the fast set-up of the system and the efficient hemodynamic support, VA-ECMO has contraindications and harbors a significant risk of complications (Table 2). Most contraindications are relative owing to the lifesaving nature of ECMO support, which in turn underlines that ECMO should only be initiated when ethical aspects or the patient’s wish do not preclude mechanical support. Uncontrolled bleeding is a contraindication, as ECMO requires heparin for anticoagulation at least for longer support. In selected patients, however, this contraindication is relative, if ECMO is the only strategy to save the life of the patient. There are indeed centers that run ECMO support in high-risk patients without any anticoagulation (off-label) for a limited time (such as in severe trauma [41] or diffuse alveolar hemorrhage [42]). A nearly absolute contraindication is severe aortic regurgitation: The retrograde flow support of VA-ECMO would cause severe LV distension and pulmonary edema. VA-ECMO results in LV distension even in patients with moderate aortic regurgitation [43]. Further contraindications are listed in Table 2.

ECMO support is an invasive procedure with profound changes of body oxygenation and circulation, and inherently associated with potentially severe complications [37, 44]. Among these are vascular complications, leg ischemia, bleeding, hyperfibrinolysis, stroke, and air embolism (Table 2). These are anticipated and in most cases effectively controlled in tertiary centers. This emphasizes that initiation, maintenance, weaning, and removal of ECMO requires a strong theoretical and practical expertise and should be performed in high-volume centers only.

### Pathophysiology: watershed

**Fig. 2:**
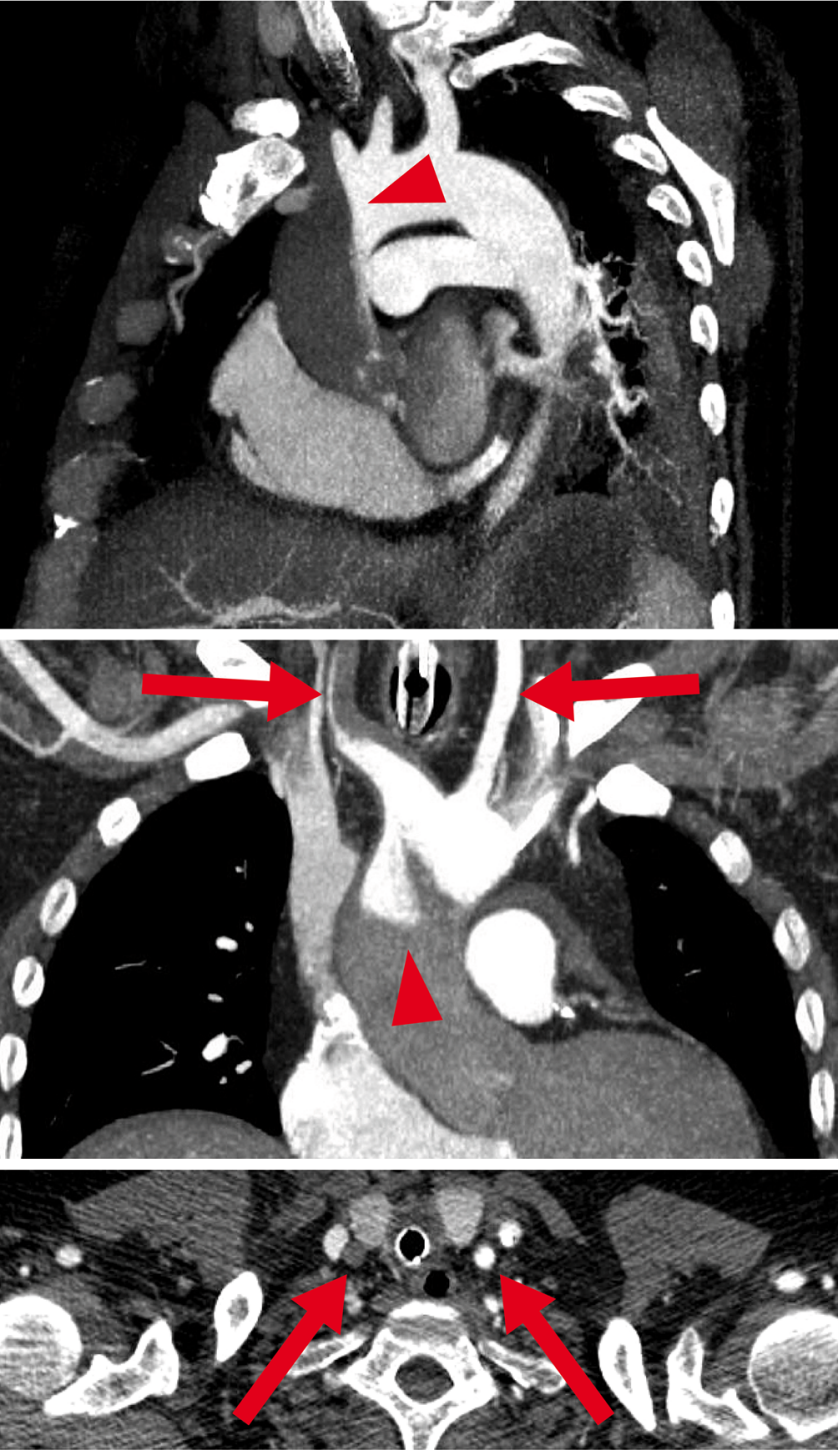
Watershed phenomenon during VA-ECMO. Computed tomography. Antegrade blood flow (*low contrast*) from the heart competes with retrograde blood flow (*high contrast*) from the ECMO in the aorta, resulting in a watershed phenomenon (*arrowhead*). Here computed tomography of a patient with pulmonary embolism and reduced cardiac output demonstrates a rather proximal watershed, leading to perfusion of the right carotid artery with “heart blood” (*dark*) and the left carotid artery with “ECMO blood” (*bright, arrows*). *Upper panel:* sagittal oblique maximum intensity projection (MIP); *middle panel:* coronal oblique MIP; *lower panel*: transverse plane. (From Napp et al. [36]; © L. C. Napp, C. Kühn, M. M. Hoeper et al. 2015. This publication is an open access publication, available on springerlink.com)

The retrograde ECMO output meets the antegrade LV output at a zone called the “watershed” [36, 45, 46]. In most cases the watershed occurs somewhere between the aortic root and the diaphragm (Fig. 2), depending on the native output of the heart: The higher the LV output relative to ECMO output, the more distal the watershed [46]. Since the output of most ECMO devices is nonpulsatile, pulse pressure measured at the right radial artery serves as an estimate of LV output [46]. For example, a blood pressure of 80/70 mm Hg at an ECMO flow of 4.5 l/min suggests a watershed in the aortic root, whereas a blood pressure of 140/70 mm Hg at the same ECMO flow suggests a watershed in the descending thoracic aorta. Blood from the ECMO is usually well oxygenated; however, oxygenation of blood from the LV depends on the respiratory function of the lung. Therefore the position of the watershed is critical for oxygenation. Aortic root oxygenation cannot be continuously measured with standard equipment. If the watershed is located in the ascending aorta and blood from the LV has an oxygen saturation of, e. g., 56% during lung failure, then the heart itself may be perfused for hours or days with an extremely insufficient oxygen saturation from the lungs in the presence of sufficient oxygenation of all other organs from the ECMO. In this context, the extreme form of dismal circulation is the “two-circulation-syndrome” [47]: If the venous cannula is incorrectly placed in the inferior caval vein, so that only blood from the lower body is drained, blood from the upper body goes through the lungs to the ascending aorta. Then venous drainage from and the perfusion of the upper body are both disconnected from that of the lower body. This results in a “Harlequin”-like appearance of the patient, with upper-body hypoxia and lower-body hyperoxia.

As outlined, circulation and oxygenation are overall subject to profound changes during VA-ECMO. Therefore multiple parameters have to be monitored in a patient on VA-ECMO at the same time (Table 3; [48]).

**Table 3:** Monitoring of patients on VA-ECMO^a^

Parameter	Reason/surrogate
**Hemodynamics**	
PA catheter: Mean PA pressure, PC wedge pressure	Efficacy of preload reduction
Central venous pressure	Efficacy of preload reduction
Right radial pulsatility	LV output
Right radial mean blood pressure	Perfusion pressure
Consider CCO catheter^b^	LV output
Central venous oxygen saturation	Systemic circulation
Urine output	Renal perfusion and function
Lab: liver enzymes	Venous decongestion
**Respiratory support**	
Right radial blood gases	Brain oxygenation, decarboxylation
Lactate	End organ ischemia
Transcutaneous continuous near-infrared spectroscopy	Tissue oxygenation (independent of pulsatility)
Pulse oximetry (right hand finger or ear)	Tissue oxygenation (largely dependent of pulsatility)
Acral perfusion (clinical)	Tissue perfusion
ECMO outflow blood gases	Control of oxygenator capacity
**Imaging**	
Echocardiography	LV distension
	Aortic regurgitation
	Pericardial effusion
	RV function
	LV thrombus
Chest X‑Ray	Pulmonary edema, pneumothorax
Pleural sonography	Pleural effusion
**Coagulation**	
D-dimer, fibrinogen, platelet count	Hyperfibrinolysis
Free hemoglobin, LDH	Hemolysis
Activated clotting time (POCT)	Anticoagulation
Blood cell count	Anemia, thrombopenia
**Leg perfusion**	
Clinical perfusion assessment	Ischemia of the cannulated leg
**General critical care monitoring**	

*CCO* continuous cardiac output, *LDH* lactate dehydrogenase, *LV* left ventricle, *PA* pulmonary artery, *PC* pulmonary capillary, *POCT* point of care testing

^a^Peripheral femoro-femoral cannulation

^b^Classic thermodilution is not reliable owing to right atrial drainage

### Triple cannulation

VA-ECMO delivers powerful circulatory and respiratory support (Table 2). Carbon dioxide elimination by the ECMO is nearly always sufficient, thus hypercapnia is nearly never a problem in patients on ECMO support – in contrast to (differential) hypoxia. As outlined earlier, the high oxygen content of ECMO output reaches only organs below the watershed. Thus, under normal conditions the lower extremities, gut, kidneys, liver etc. are well oxygenated during VA-ECMO support. An additional effect on organ oxygenation results from a higher amount of oxygen delivered to the lower body and an associated higher venous backflow oxygen: Depending on oxygenation settings, ECMO outflow pO2 usually equals at least 200–300 mm Hg, compared with 50–100 mm Hg in arterial blood oxygenated in the lungs of a standard ventilated shock patient. This results in a higher total oxygen delivery to the body, which may have an effect also on organs perfused by LV blood, yet the relevance of this effect is unclear to date.

**Fig. 3:**
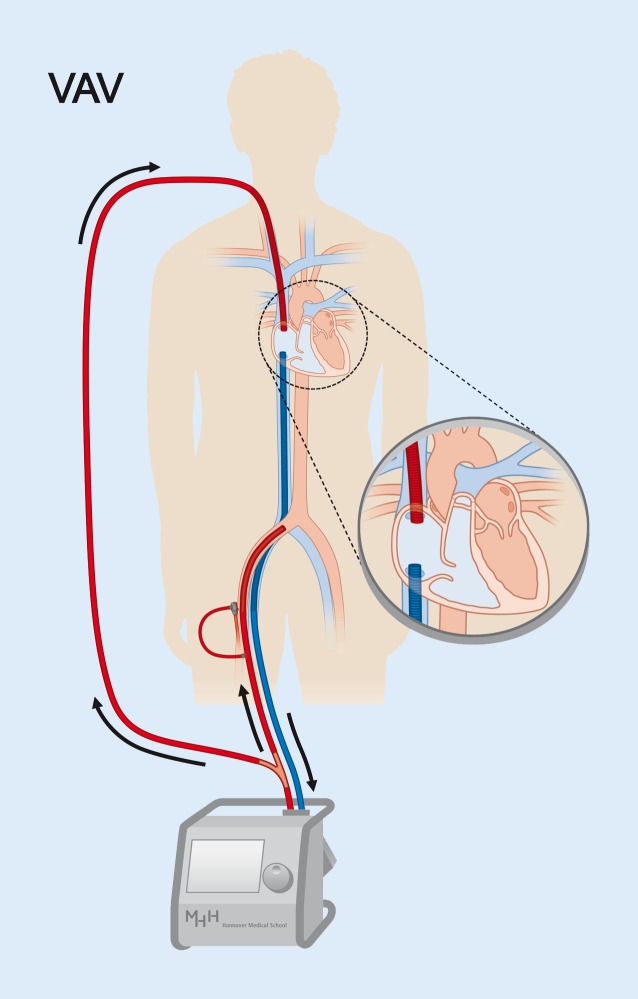
Veno-arterial-venous (*VAV*) ECMO. VAV-ECMO drains venous blood (*blue*) from the right atrium and returns balanced volumes of blood after reoxygenation and decarboxylation (*red*) to the iliac artery toward the aorta and to the right atrium toward the pulmonary circulation. For this purpose, the ECMO outflow is divided by a Y-connector. Flow through the returning cannulae is balanced with an adjustable clamp and monitored with a separate flow sensor on the upper return cannula. (Modified from Napp & Bauersachs [49]; © L. C. Napp, J. Bauersachs 2016. This publication is an open access publication, available on intechopen.com)

However, in some patients on VA-ECMO support secondary lung failure develops. This is a dangerous situation: Depending on the watershed position, all organs perfused by blood from the heart are prone to severe ischemia in the presence of ECMO support, in particular the heart and brain. If lung failure is due to pulmonary edema, ultrafiltration and active LV unloading (see later) are sufficient to achieve decongestion. However, in many patients with lung failure on VA-ECMO support, the problem results from an ARDS-like condition, which cannot be or should not be effectively solved by aggressive ventilation or decongestion. In these patients an elegant and very effective treatment is upgrading the ECMO circuit to a triple-cannulated ECMO, with one venous-draining, one arterial-supplying, and one venous-supplying cannula (“VAV-ECMO”, Fig. 3; [36, 49]). In addition to the VA circuit, the additional venous cannula adds preoxygenated blood to the lungs and thereby establishes a “VV component.” This ensures sufficient oxygen content of blood ejected by the heart and allows for lung protective ventilation. Of note, VAV-ECMO requires sufficient RV function, otherwise it may be necessary to relocate the venous-supplying cannula into the pulmonary artery [49] for bypassing the RV. Retrospective studies suggest efficacy of VAV cannulation for rescue of body oxygenation and recovery of lung failure [50–52], but prospective studies are needed to confirm the observed benefit.

### Pathophysiology: afterload, decompression

During acute heart failure, the diseased LV has impaired ability to eject, and stroke work and myocardial oxygen consumption are increased [30, 53]. When bridge-to-recovery is the therapeutic goal (e. g., myocarditis or myocardial infarction), stroke work and myocardial oxygen consumption have to be reduced to facilitate regeneration. However, notwithstanding the immediate massive hemodynamic and respiratory support and the reduction of preload, VA-ECMO increases LV afterload [53–57]. This may result in increased LV filling pressures, wall stress, and severe pulmonary congestion despite reduction of preload. Moreover, ECMO is often ascribed a positive effect on coronary perfusion; however, human data are lacking and data from animal studies are conflicting [58, 59]. From a pathophysiological perspective, a high LV pressure during diastole impairs coronary perfusion by reducing the transcoronary perfusion gradient. In patients with extremely low systolic LV function and in all patients with ongoing arrest, VA-ECMO support results in a functionally closed aortic valve without relevant transaortic blood flow. This potentially results in severe LV distension [54] and pulmonary congestion in the presence of sufficient systemic circulation.

**Fig. 4:**
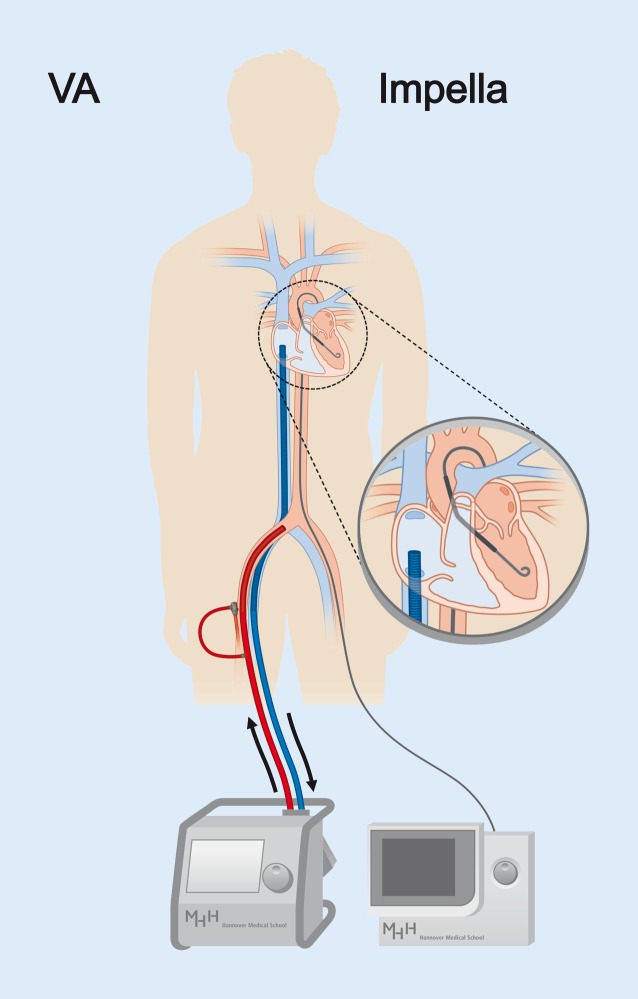
VA-ECMO and active LV unloading by using an Impella® microaxial pump. In addition and in contrast to VA-ECMO, which delivers retrograde flow support to the aorta, the Impella® pump drains the LV and supplies the blood to the ascending aorta. This “unloads” the LV and facilitates myocardial recovery and pulmonary decongestion. (Modified from Napp & Bauersachs [49]; © L. C. Napp, J. Bauersachs 2016. This publication is an open access publication, available on intechopen.com)

Thus, LV unloading, prevention of LV distension, reduction of myocardial wall stress, and enhancement of coronary perfusion are important goals during mechanical circulatory support for bridge-to-recovery. Unloading (= “venting”) can be achieved by different methods. One way is venting through the atrial septum, either by atrioseptostomy [60, 61] or placement of an additional draining cannula through the atrial septum [62], both of which are potentially hazardous [61] particularly in the already critically ill patient. Another possibility is transvalvular unloading across the aortic valve, which has already been performed in an experimental approach with a transvalvular coronary catheter connected to the venous draining ECMO cannula [63]. However, simple draining of the LV has no direct effect on coronary perfusion and does not increase antegrade transaortic blood flow. Therefore pumps have been developed that are percutaneously inserted, drain the LV, and eject into the ascending aorta. Their first use (Hemopump®) was published as early as in 1990 [64], but the clinical breakthrough took nearly 20 years to occur, mainly attributed to technical improvement of the device. Today, the only transvalvular microaxial pump approved in the United States and Europe is the Impella® device (Abiomed, Danvers, USA), which is the current device of choice of most centers for active LV unloading, also combined with VA-ECMO (Fig. 4).

**Fig. 5:**
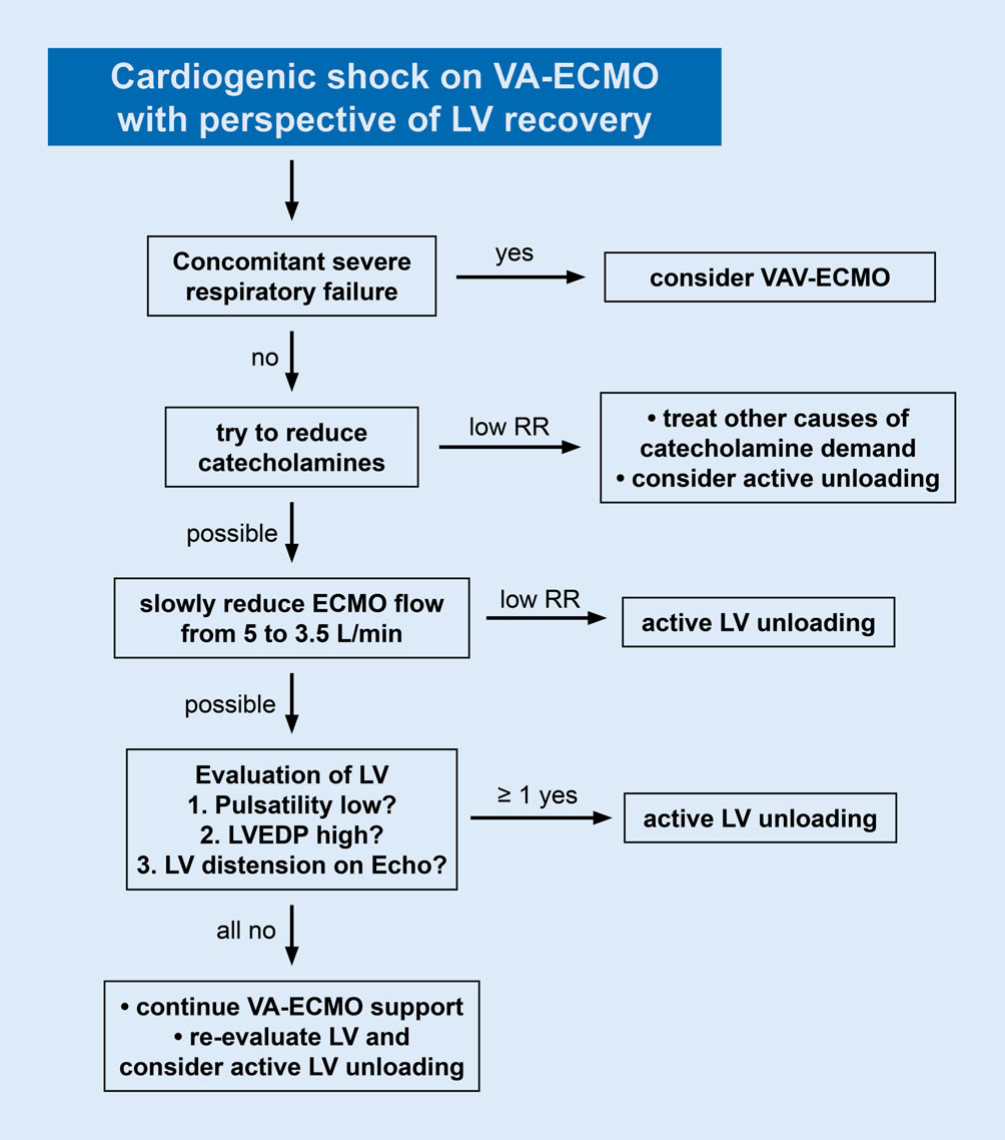
Management of VA-ECMO for bridge-to-recovery in cardiogenic shock. Proposal of mechanical support strategies for patients with cardiogenic shock and prospect of cardiac recovery. *LVEDP* left ventricular end-diastolic pressure, *RR* arterial blood pressure, *VAV-ECMO* venoarteriovenous extracorporeal membrane oxygenation

The frequency of the combined use of ECMO and Impella® varies greatly between centers. Of note, it is unclear to date which patients have a benefit of additional Impella® support in parallel to VA-ECMO. There are two published studies reporting combined support [65, 66]. Their data point to a benefit of dual support, but further studies are unequivocally needed. Fig. 5 shows a proposal for the management of VA-ECMO and potential unloading, based on pathophysiological considerations and clinical practice in our center. In general, the lower systolic LV function is in a given patient, the sooner active LV unloading should be considered.

### VA-ECMO for cardiogenic shock

Despite the broad use of ECMO in experienced centers, data from larger studies are limited. Most studies are retrospective series or registry studies. Some years ago, IABP was used in many countries almost routinely for patients with severe cardiogenic shock, but later on randomized studies demonstrated the noneffectiveness of routine IABP support [33]. With this in mind, the decision for or against mechanical support and the decision for a specific device should take into account several different factors such as RV and LV function, valve status, and lung function. The available devices (ECMO, Impella®, TandemHeart®) each have unique features, and there is no uniform device covering all types of cardiogenic shock. This is one of the major limitations of nearly all retrospective studies.

From clinical experience, ECMO initiation is rather easy and fast, and ECMO is a very effective tool for enhancing and ensuring systemic circulation and provide gas exchange. As such, it should be primarily considered in patients with severe acute cardiorespiratory failure (the “crash and burn” patient). In addition, some specific indications exist, such as decompensated pulmonary arterial hypertension and pulmonary embolism. Table 4 lists selected studies [67–72] of VA-ECMO in cardiogenic shock.

**Table 4:** Selected studies of VA-ECMO for cardiogenic shock

Reference	Origin	Design	Comparison	Etiology	Patients (*N*)	Age	Implantation	LVEF	Outcome	Complications
Sheu et al. [67]	Taiwan	Prospective observational	ECMO+IABP vs. IABP	100% STEMI in both groups	46 vs. 25 sex not reported	65.1± 10.6 years vs. 67.2± 11.1 years (mean, SD)	In the cathlab (probably shortly after PCI, but timepoint not exactly reported)	Data not reported	30 d-survival 60.9% ECMO-IABP vs. 28.0% IABP	Bleeding or vascular complications 39.1%
Tsao et al. [68]	Taiwan	Retrospective	ECMO+IABP vs. IABP	ECMO+IABP: 54.5% STEMI, 45.5% NSTEMI (93.9% had IABP) IABP: 44.0% STEMI, 56.0% NSTEMI (100% had IABP)	33 vs. 25 84.8% vs. 64.0% men	74.1 ± 12.2 years vs. 70.1 ± 17.0 years (mean, SD)	In the emergency room or cathlab	ECMO+IABP: 38 ± 10% IABP: 39 ± 14%	Successful weaning 81.8% in ECMO+IABP vs. 44.0% in IABP survival to discharge 66.7% in ECMO+IABP vs. 32.0% in IABP 1‑year survival 63.6% in ECMO+IABP vs. 24.0% in IABP	Data not reported
Sakamoto et al. [69]	Japan	Retrospective	no device comparison all had VA-ECMO	100.0% ACS, 36.7% had cardiac arrest before ECMO 95.9% received emergency revascularization	98 66.3% men	72 ± 12 years (mean, SD)	44.9% implant on admission, 33.7% implant during PCI, 20.4% implant after PCI. 95.9% had additional IABP	Data not reported	Successful weaning 55.1% survival to discharge 32.7%	35.7% ECMO-related complications 23.5% cannula site complications 4.1% retroperitoneal hemorrhage 7.1% lower limb ischemia 3.1% cerebral hemorrhage
Sattler et al. [70]	Germany	Retrospective	ECMO vs. IABP	ECMO: 66.7% STEMI, 33.3% NSTEMI, with 66.7% OHCA and 16.7% IHCA IABP: 83.3% STEMI, 16.7% NSTEMI, with 41.7% OHCA and 16.7% IHCA	12 vs. 12 83.3% men in both groups	54.8 ± 13.3 years vs. 68.3 ± 12.2 years (mean, SD)	1 pat. before PCI 9 pat. immediately after PCI 2 pat. 24 and 48 h after PCI and IABP	ECMO: 48 ± 10% IABP: 32 ± 13%	30 d-survival 67.0% ECMO vs. 33.0% IABP	3/12 bleeding 2/12 compartment syndrome hemolysis with 21.0 ± 12.4 packed red blood cell transfusions per patient
Aso et al. [71]	Japan	Register	no device comparison all had VA-ECMO	42.2% Ischemic heart disease (IHD), 34.8% Heart failure (HF), 13.7% Valvular heart disease (VHD), 4% Myocarditis (MYO), 4.1% Cardiomyopathy (CMP), 0.7% Takotsubo syndrome (TS), 0.3% Infectious endocarditis (IE) Patients who had cardiac arrest: All 47%, IHD 25.0%, HF 15.0%, VHD 2.7%, MYO 1.4%, CMP 2.5%, TS 0.3%, IE 0.06%	4,658 73.0% men	All 64.8 ± 13.7 years (mean, SD)	Data not reported 60.8% had IABP prior to or in parallel to VA-ECMO	Data not reported	Survival to discharge all patients 26.4%, IHD 20.9%, HF 32.2%, VHD 23.0%, MYO 43.0%, CMP 26.9%, TS 35.3%, IE 25.0%	Data not reported
Muller et al. [72]	France	Prospective observational	no device comparison all had VA-ECMO	100% acute myocardial infarction 13.8% received VA-ECMO during CPR and 43.5% after CPR	138 79.7% men	55 (46–63) years (median, IQR)	10.1% before and 89.9% after PCI 69.6% had IABP parallel to ECMO 2.2% had Impella and ECMO 11.6% were switched to central ECMO cannulation	20 (15–25)% (median, IQR)	Successful weaning 35.5% 6-months survival 41.3%	39.1% ECMO complications: 12.3% bleeding 10.9% leg ischemia 11.6% access site infection 3.6% hemolysis 11.6% overt pulmonary edema on ECMO

*CPR* cardiopulmonary resuscitation, *ECMO* extracorporeal membrane oxygenation, *ECPR* extracorporeal CPR, *IABP* intra-aortic balloon pump, *IQR* interquartile range, *LVEF* left ventricular ejection fraction, *NSTEMI* Non-ST-elevation myocardial infarction, *pat*. patients,* PCI* percutaneous coronary intervention, *STEMI* ST-elevation myocardial infarction

From a pathophysiological perspective, VA-ECMO should be favored for bridge-to-destination or bridge-to-transplantation, when recovery is not the primary goal and LVAD or transplantation will follow. VA-ECMO is also favorable for bridge-to-surgery, especially for embolectomy. In resuscitated patients VA-ECMO is the commonly used device for bridge-to-decision. By contrast, VA-ECMO may not be the ideal support form for isolated LV dysfunction with potential for recovery (acute myocardial infarction, myocarditis, Takotsubo syndrome, etc.), since afterload increases and recovery may be hampered [53]. Of note, these are considerations from daily clinical routine and pathophysiology, but dedicated studies are urgently needed to prospectively compare the different support forms. One such study is the prospective, open-label, multicenter, randomized, controlled “ANCHOR” trial (*A*ssessment of ECMO in Acute Myocardial Infarction with *N*on-reversible *C*ardiogenic Shock to *H*alt *O*rgan Failure and *R*educe Mortality), which is currently investigating the use of ECMO in cardiogenic shock during myocardial infarction. In this context, an interesting tool that is already mentioned in current heart failure guidelines [31] is the “SAVE” score to estimate the prognosis of patients with cardiogenic shock on VA-ECMO [73]. Another promising score is the “ENCOURAGE” score [72].

### VA-ECMO for extracorporeal resuscitation

Table 5 lists a selection of studies [74–86] on extracorporeal CPR (ECPR), i. e., ECMO for refractory resuscitation. Of note, to date there is no prospective randomized study on ECMO for this indication, also for ethical reasons. A comprehensive review of retrospective studies has been recently published elsewhere [87]. Taken together, the available literature on ECPR suggests that ECMO is sufficient to ensure systemic circulation in refractory arrest. However, mortality varies between centers, and four factors appear to critically determine ECPR success: patient selection criteria, a detailed standard operating procedure, immediate and sufficient bystander CPR, and time from arrest to ECMO. Table 6 lists a proposal for inclusion and exclusion criteria for ECPR. Of note, such criteria can only set a frame for decision, but may need to be adjusted for individual patients. A standard operating procedure for ECPR needs to incorporate all elements from circulatory arrest and bystander CPR over professional CPR, early contact with the ECMO center, team approach by anesthesiologists, cardiologists, and intensivists, high-level intensive care medicine, and optimal rehabilitation. A proposal for a prospective study considering all these factors has recently been published [88]. The time-to-ECMO interval is consistently associated with mortality [78, 84], very likely due to the increased incidence and severity of post-resuscitation metabolism with delayed extracorporeal support. Thus, a dedicated program for ECPR needs to put all efforts into earliest ECMO implantation and optimal preclinical CPR.

**Table 5:** Selected studies of VA-ECMO for cardiac arrest

Reference	Origin	Design	IHCA/OHCA	Etiology	Patients (*N*)	Age	Bystander CPR	Initial rhythm	Time-to-ECMO	Initial pH	Initial lactate	Outcome	ECMO-related complications	Predictors of mortality
Chen et al. [74]^a^	Taiwan	Retrospective	96.5%/3.5%	24.6% post cardiotomy all cardiac origin, further details not reported	57 59.6% men	57.1 ± 15.6 years (mean, SD)	96.5%	VF 47.4%, VT 14.0%, PEA/asystole 38.6%	47.6 ± 13.4 min. (mean, SD)	Data not reported	Data not reported	Weaning off ECMO 66.7% overall survival 31.6% post-cardiotomy 57.1% non-post-cardiotomy 23.3%	Massive retroperitoneal hematoma 1.8% limb amputation after ECMO cannulation 1.8% further data not reported	Aspartate aminotransferase on day 3 lactate on day 3
Massetti et al. [75]	France	Retrospective	87.5%/12.5%	40% ACS, 10% HF, 15% Intoxication, 10% RHY, 10% post-cardiotomy, 7.5% PE, 5% MYO	40 57.5% men	42 ± 15 years (mean, SD)	Data not reported	Data not reported	105 ± 44 min. (mean, SD)	Data not reported	Data not reported	Weaning off ECMO 30% survival to discharge 20%	Vascular complications 12.5% leg ischemia 2.5% bleeding 7.5%, pulmonary hemorrhage 12.5%	Time-to-ECMO
Sung et al. [76]	South Korea	Observational	100%/0%	36.3% coronary artery disease, 36.3% after cardiac surgery, 9% HF, 9% others, 4.5% PE, 4.5% MYO	22 54.5% men	62.5 ± 14.0 years (mean, SD)	Data not reported	Data not reported	48.5 ± 29.0 min. (mean, SD)	Data not reported	Data not reported	Weaning off ECMO 59.1% survival to discharge with good neurological outcome 40.9%	13.6% bleeding 4.5% vascular complications	Data not reported
Chen et al. [77] ^a^	Taiwan	Prospective observational	100%/0%	62.7% ACS, 10.2% HF, 8.5% MYO, 11.9% post-cardiotomy, 1.7% PE, 5.1% others	59 84.7%	57.4 ± 12.5 years (mean, SD)	Data not reported (although 100% witnessed arrest)	VT/VF 49.2%, PEA 28.8%, Asystole 22.0%	52.8 ± 37.2 min. (mean, SD)	Data not reported	Data not reported	Weaning off ECMO 49.2% survival to discharge 28.8% 1-year survival 18.6%	Data not reported	Time-to-ECMO initial rhythm other than VT/VF
Kagawa et al. [78]	Japan	Retrospective IHCA vs. OHCA	49.4%/50.6%	IHCA 55% ACS, 3% HF, 5% MYO, 16% PE, 21% others OHCA 56% ACS, 5% HF, 3% MYO, 15% PE, 21% others	38 vs. 39 58%/85% men	68 (58–73) years vs. 56 (49–64) years (median, IQR)	92% in IHCA 72% in OHCA	IHCA VT/VF 26%, PEA 68%, Asystole 5% OHCA VT/VF 49%, PEA 36%, Asystole 15%	IHCA 25 (21–43) min. OHCA 59 (45–65) min. (median, IQR)	IHCA 7.24 (7.09–7.39) OHCA 7.02 (6.90–7.14) (median, IQR)	Data not reported	Weaning off ECMO IHCA 61%, OHCA 36% good neurological outcome at discharge IHCA 26%, OHCA 10% 30-days survival IHCA 34%, OHCA 13%	leg ischemia IHCA 18%, OHCA 21% Bleeding or hematoma IHCA 68%, OHCA 59%	Time-to-ECMO initial rhythm other than VF
Le Guen et al. [79]	France	Prospective observational	0%/100%	86% cardiac (no further details), 6% trauma, 4% drug overdose, 2% respiratory, 2% others	51 90% men	42 ± 15 years (mean, SD)	Data not reported	VF 63%, Asystole 29%, PEA 8%	120 (102–149) min. (median, IQR)	6.93 ± 0.17 (mean, SD)	19.9 ± 6.7 (mean, SD)	24 h-survival 40% 48 h-survival 12% survival with good neurological outcome at day 28 4%	14% severe hemorrhage further data not reported	Lactate at baseline end-tidal CO2 time-to-ECMO
Avalli et al. [80]	Italy	Retrospective IHCA vs. OHCA	57.1%/42.9%	IHCA 37% ACS, 33% post cardiotomy, 13% PE, 9% HF, 9% others OHCA 67% ACS, 5% HF, 11% RHY, 17% others	24 vs. 18 67%/94% men	67 (61–73) years vs. 46 (37–64) years (median, IQR)	IHCA 100% OHCA 55%	IHCA VT/VF 50%, PEA/Asystole 50% OHCA VT/VF 89%, PEA/Asystole 11%	IHCA 55 (40–70) min. OHCA 77 (69–101) min. (median, IQR)	Data not reported	Data not reported	Weaning off ECMO IHCA 58%, OHCA 16% 28-days survival IHCA 46%, OHCA 5%	IHCA 46% vascular compl. OHCA 33% vascular compl.	Data not reported
Chung et al. [81]	Taiwan	Prospective observational	100%/0%	27.6% STEMI, 11.9% NSTEMI, 22.4% post-surgery, 10.5% HF, 19.4% MYO, 6.0% post-PCI, 2.2% others	134 77.6% men	51.8 ± 20.5 years (mean, SD)	100%	VT/VF 27.6%, further data not reported	Data not reported	Data not reported	Data not reported	Weaning off ECMO 50.7% survival to discharge 42.5% survival 30 days 54.5%	Overall 21.6% peripheral limb ischemia 3.0% further data not reported	APACHE-II-Score ≥22 unsuccessful weaning off ECMO
Haneya et al. [82]	Germany	Retrospective	69.4%/30.6%	30.6% ACS, 15.3% HF, 17.6% post-PCI/TAVI, 16.5% PE, 2.4% HYPO, 5.9% TRA, 11.6% others. Post-cardiotomy patients were excluded	85 71.8% men	59 ± 16 years (mean, SD)	Data not reported	VT/VF 29.4%, PEA 42.4%, Asystole 28.2%	51 ± 35 min. (mean, SD)	All 7.01 ± 0.22 IHCA 7.09 ± 0.18 OHCA 6.85 ± 0.24 (mean, SD)	All 11 ± 6.9 IHCA 7.2 ± 5.6 OHCA 14.7 ± 9.1 (mean, SD)	Weaning off ECMO 47.1% (IHCA 57.6%, OHCA 23.1%) survival to discharge 34.1% (IHCA 42.4%, OHCA 15.4%) 93.1% without severe neurological deficit among discharged patients	Overall 32.9% leg ischemia 16.5% bleeding 3.5% cannulation complications 12.9%	pH, CPR duration
Fagnoul et al. [83]	Belgium	Prospective observational	41.7%/58.3%	29.2% ACS, 20.8% RHY, 12.5% PE, 8.3% TRA, 8.3% Intoxication, 12.5% HYPO, 8.3% others	24 58.3% men	48 (38–55) years (median, IQR)	91.7%	VT/VF 41.7%, PEA/Asystole 58.3%	58 (45–70) min. (median, IQR)	Survivors 7.22 ± 0.23 non-survivors 7.06 ± 0.22 (mean, SD)	Survivors 9.8 ± 5.3 non-survivors 14.9 ± 4.85 (mean, SD)	Weaning off ECMO 29.2% survival to ICU discharge 25.0%	Major bleeding on ECMO site 29.2% diffuse bleeding 41.7%	Time-to-ECMO (non-significant trend)
Leick et al. [84]	Germany	Retrospective	0%/100%	53.6% ACS, 21.4% HF, 23.1% septic shock, 7.1% Takotsubo syndrome, 3.6% PE, 3.6% MYO	28 53.6% men	53.9 ± 15.9 years (non-survivors) 60.3 ± 9.6 years (survivors) (mean, SD)	Data not reported	VF 28.6%, Asystole 21.4%, PEA 39.3%, 10.7% not reported	44.0 (31.0–45.0) min. (survivors) 53.0 (40.0–61.3) min. (non-survivors) (median, IQR)	Survivors 7.2 (7.05–7.4) non-survivors 7.1 (7.0–7.3) (median, IQR)	Survivors 4.5 (3.9–9.3) non-survivors 4.7 (3.6–7.8) (median, IQR)	30-day survival 39.3%	leg ischemia 3.6% bleeding 32.1%	Time-to-ECMO
Stub et al. [85]	Australia	Prospective observational	57.7%/42.3%	53.8% ACS, 7.7% HF, 11.5% Arrhythmia, 7.7% PE, 7.7% respiratory, 11.5% others	26 77% men	52 (38–60) years (median, IQR)	Data not reported	VF 73.1%, PEA 15.4%, Asystole 11.5%	56 (40–85) min. (median, IQR)	all 6.9 (6.7–7.1) survivors 7.0 (6.8–7.1) non-survivors 6.8 (6.7–7.0) (median, IQR)	all 10 (7–14) survivors 8 (6–12) non-survivors 13 (9–14) (median, IQR)	Weaning off ECMO 54.1% survival to discharge 53.8%	Bleeding 69.2% peripheral vascular issues 38.5% vascular surgery 41.7%	Time-to-ECMO, pH, troponin
Jung et al. [86]	Germany	Retrospective	70.9%/29.1%	23.1% VT/VF in HF, 40.2% VT/VF in ACS, 28.1% post-surgery/-intervention, 9.4% others	117 68.4% men	61 (51–74) years (median, IQR)	Data not reported	VT/VF 63.2%, further data not reported	Data not reported	Data not reported	all 9.0 (4.5–14.5) survivors 4.5 (2.9–6.2) non-survivors 11.7 (5.5–14.9) (median, IQR)	Weaning off ECMO 52.1% 30-days survival 23.1% good neurological outcome 14.5%	Data not reported	Lactate, hemoglobin

*ACS* acute coronary syndrome, *CPR* cardiopulmonary resuscitation, *ECMO* extracorporeal membrane oxygenation, *HF* heart failure, *HYPO* accidental hypothermia, *IHCA* in-hospital cardiac arrest, *IQR* interquartile range, *MYO* myocarditis, *NSTEMI* non-ST-elevation myocardial infarction, *OHCA* out-of-hospital cardiac arrest, *PE* pulmonary embolism, *PEA* pulseless electrical activity, *RHY* arrhythmia, *SD* standard deviation, *STEMI* ST-elevation myocardial infarction, *TRA* trauma, *VF* ventricular fibrillation, *VT* ventricular tachycardia

^a^No overlapping patients

**Table 6:** Proposed criteria for extracorporeal CPR (ECPR)

**Inclusion criteria (all need to be met)**
Witnessed circulatory arrest
Bystander CPR
Age <75 years^a^
No ROSC after 10 min of professional CPR^b^
**Exclusion criteria (one criterion is sufficient)**
Severe comorbidity (cancer, end-stage liver cirrhosis, etc.)
Preexisting cognitive impairment/brain damage
Preclinical CPR >1h^c^
**Optional exclusion criteria**
pH at baseline <6.8
Lactate at baseline >15 mmol/l
**Exceptions for criteria above**
Accidental hypothermia

*CPR* cardiopulmonary resuscitation, *ECMO* extracorporeal membrane oxygenation

^a^Age limit depends on comorbidities and biological age

^b^Excellent CPR until ECMO is an essential prerequisite for success

^c^May be extended in single cases, when very young patients need time for transfer and have optimal CPR

### Conclusion

Mechanical support is increasingly used in cardiogenic shock to minimize or avoid catecholamines and to facilitate regeneration of the diseased heart. Refractory cardiac arrest is an emerging indication for mechanical support, and recently more centers have developed ECPR programs. Cardiogenic shock and arrest share many pathophysiological features, and in this context VA-ECMO is a powerful extracorporeal life support system, as long as it is initiated early. VA-ECMO use requires a dedicated bridging strategy, such as bridge-to-recovery, bridge-to-decision, or bridge-to-destination, and complications need to be anticipated. Retrograde flow support increases LV afterload and may result in LV distension, which can be prevented and resolved by LV venting or active LV unloading. Prospective controlled studies are needed to develop specific protocols for defined clinical conditions, in order to find the optimal mechanical support strategy in a given situation.

## Abbreviations

CPR: Cardiopulmonary resuscitation
ECMO: Extracorporeal membrane oxygenation
ECPR: Extracorporeal cardiopulmonary resuscitation
IABP: Intra-aortic balloon pump
LV: Left ventricle
LVAD: Left ventricular assist device
OHCA: Out-of-hospital cardiac arrest
ROSC: Return of spontaneous circulation
